# Mitochondrial genome sequencing helps show the evolutionary mechanism of mitochondrial genome formation in *Brassica*

**DOI:** 10.1186/1471-2164-12-497

**Published:** 2011-10-11

**Authors:** Shengxin Chang, Tiantian Yang, Tongqing Du, Yongjuan Huang, Jianmei Chen, Jiyong Yan, Jianbo He, Rongzhan Guan

**Affiliations:** 1 State Key Laboratory of Crop Genetics and Germplasm Enhancement, Nanjing Agricultural University, Nanjing 210095, PR China; 2 Institute of Vegetable Crops, Jiangsu Academy of Agricultural Sciences, Nanjing 210014, PR China

## Abstract

**Background:**

Angiosperm mitochondrial genomes are more complex than those of other organisms. Analyses of the mitochondrial genome sequences of at least 11 angiosperm species have showed several common properties; these cannot easily explain, however, how the diverse mitotypes evolved within each genus or species. We analyzed the evolutionary relationships of *Brassica *mitotypes by sequencing.

**Results:**

We sequenced the mitotypes of *cam *(*Brassica rapa*), *ole *(*B. oleracea*), *jun *(*B. juncea*), and *car *(*B. carinata*) and analyzed them together with two previously sequenced mitotypes of *B*. *napus *(*pol *and *nap*). The sizes of whole single circular genomes of *cam*, *jun*, *ole*, and *car *are 219,747 bp, 219,766 bp, 360,271 bp, and 232,241 bp, respectively. The mitochondrial genome of *ole *is largest as a resulting of the duplication of a 141.8 kb segment. The *jun *mitotype is the result of an inherited *cam *mitotype, and *pol *is also derived from the *cam *mitotype with evolutionary modifications. Genes with known functions are conserved in all mitotypes, but clear variation in open reading frames (ORFs) with unknown functions among the six mitotypes was observed. Sequence relationship analysis showed that there has been genome compaction and inheritance in the course of *Brassica *mitotype evolution.

**Conclusions:**

We have sequenced four *Brassica *mitotypes, compared six *Brassica *mitotypes and suggested a mechanism for mitochondrial genome formation in *Brassica*, including evolutionary events such as inheritance, duplication, rearrangement, genome compaction, and mutation.

## Background

Plant mitochondrial genomes are complex because they encode significantly more genes than do their fungal and animal counterparts. Investigations of the mitochondrial genome sequences of at least 13 angiosperm species, including *Arabidopsis thaliana *[1], *Beta vulgaris *[2], *Oryza sativa *[3-5], *Brassica napus *[6,7], *Zea mays *[8-10], *Nicotiana tabacum *[11], *Triticum aestivum *[12], *Vitis vinifera *[13], *Citrullus lanatus *and *Cucurbita pepo *[14], and *Vigna radiata *[15], together with physical mapping [16-18], have showed several properties of plant mitochondrial genomes, such as large size (200-2400 kb), slow rates of evolutionary change, incorporation of foreign DNA, a multipartite structure, and specific modes of gene expression (e.g. *cis *and *trans *splicing, RNA editing), etc [19]. These properties cannot easily explain how the diverse mitotypes evolved within each plant genus or species. To understand the evolutionary peculiarity of plant mitotypes, which are defined as mitochondrial genome types of which there can be more than one within one plant species or genus, more systematic sequences are needed. To date, no systematic sequences of one angiosperm genus with multiple species had been used to analyze the derivation of mitochondrial genome, and thus the mechanism underlying the peculiarity has not been revealed. Here, we selected *Brassica *as an evolutionary clade in which to analyze the evolutionary relationships of *Brassica *mitotypes by sequencing.

**Figure 1:**
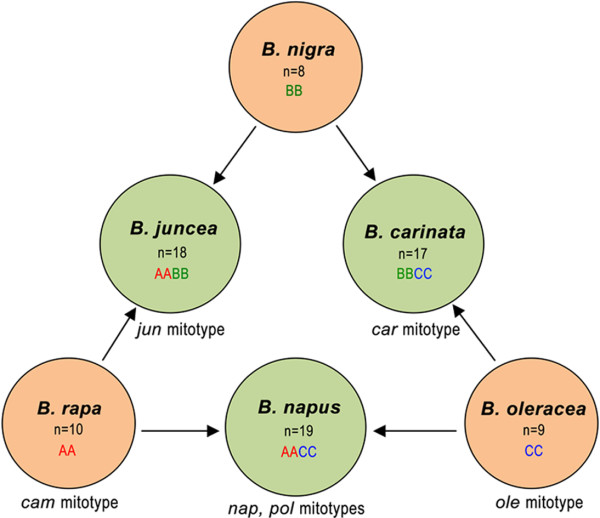
**Cytogenetic relationships of six cultivated Brassica species as depicted by U's triangle **[20]. U's triangle illustrates the evolutionary relationship between three cultivated elementary species (*B. rapa*, *B. oleracea*, and *B. nigra*) and three amphiploid species (*B. napus, B. juncea*, and *B. carinata*). Chromosome numbers, nuclear genome types and mitotypes are shown inside or outside the circle for each species.

*Brassica *contains six cultivated species that are very important for producing vegetables and oilseeds. The nuclear genomic relationships between the six species were showed by the U with cytogenetics approach [20], which has been popularly accepted as U's triangle theory (Figure 1). However, the cytoplasmic relationship between the six species needs to be further explored, although the entire sequences of two mitotypes of *Brassica napus *have been reported [6,7] with a focus on the mechanism of cytoplasmic male sterility (CMS), and mitochondrial DNA (mtDNA) of three *Brassica *species has been physically mapped [18]. Here, we report the sequence of the mitochondrial genomes of *B. rapa *(*cam *mitotype), *B. oleracea *(*ole*), *B. juncea *(*jun*), and *B. carinata *(*car*). Together with previously reported mitochondrial genome sequences of the *pol *and *nap *mitotypes in *B. napus *[6,7], we compared the six mitochondrial genome sequences to learn the mechanism of formation for *Brassica *mitochondrial genomes.

## Results

### *Brassica *mitochondrial genomes

*Brassica *comprises six cultivated species [21]: *B. rapa *(*cam*), *B. oleracea *(*ole*), *B. napus*, *B. juncea *(*jun*), *B. nigra*, and *B. carinata *(*car*). The sizes of entire single circular mitochondrial genomes of *cam *[GenBank: JF920285], *jun *[GenBank: JF920288], *ole *[GenBank: JF920286], and *car *[GenBank: JF920287] are 219,747 bp, 219,766 bp, 360,271 bp, and 232,241 bp, respectively. The mitochondrial genomes of *car *and *ole *are larger than these of *cam*, *jun*, *pol*, and *nap*. The *ole *mitotype is larger because of the duplication of a 141.8 kb segment. The G+C content of the six mitotypes ranges from 45.19% in *nap *to 45.33% in *car*, with slight differences among mitotypes. Similarly, nucleotide base content varies slightly in the six mitotypes. The total length of genes with known functions differs between the mitotypes. The percentages that the total length of genes with known functions accounts for the mitochondrial genomes are almost the same in the *cam*, *jun*, *ole*, *pol*, and *nap *mitotypes, but the percentage in *car *is 27.98% (Table 1), less than that of the other five mitotypes, perhaps as a result of differentiation of *Brassica *mitotypes.

**Table 1:** Gene contents and total length of six mitotypes

Feature	*cam*(%)	*jun*(%)	*ole*(%)	*car*(%)	*pol*(%)	*nap*(%)
Total gene content	54	54	95	53	56	55
Protein-coding genes	34	34	56	33	35	35
*rRNA*	3	3	4	3	3	3
*tRNA*	18	18	35	17	18	17
Total gene length	67729(30.82)	67732(30.82)	112418(31.20)	64976(27.98)	68408(30.62)	68415(30.84)
Protein exons	31620(14.39)	31620(14.39)	52753(14.64)	30297(13.05)	32295(14.46)	32259(14.54)
Protein introns	29590(13.47)	29593(13.47)	48722(13.52)	28234(12.16)	29594(13.25)	29711(13.39)
*rRNA*	5143(2.34)	5143(2.34)	8263(2.29)	5143(2.21)	5143(2.30)	5143(2.32)
*tRNA*	1376(0.63)	1376(0.63)	2680(0.74)	1302(0.56)	1376(0.62)	1302(0.59)

The percentage of gene length accounting for the entire genome is indicated in parentheses. *Cam*, *jun*, *ole*, and *car *denote the mitotype of *B. rapa*, *B. juncea*, *B. oleracea*, and *B. carinata*, respectively. And the *pol *and *nap *denote two mitotypes of *B. napus*.

Analysis of genes with known functions showed that these six mitotypes share 36 species of protein-encoding genes, 3 species of ribosome genes, and 15 species of tRNA genes (Table 2); paralogous genes present in more than one copy are counted here as one species. However, the *ole *and *car *mitotypes lack the complex-IV-related *cox2-2 *gene found in the other four mitotypes and the CMS-related genes (homologous to *orf224 *and *orf222*) found in the *pol *and *nap *mitotypes [22,23]. The *cam *and *jun *mitotypes that have identical gene constitution, also lack the CMS-related genes (*orf224 *and *orf222*). The numbers of genes with known functions are almost the same in all six mitotypes, but the total number of genes varies with mitotype, ranging from 53 in *car *to 95 in *ole *(Table 2).

**Table 2:** Gene contents of *Brassica *mitotypes

Product group	Gene	*cam*	*jun*	*pol*	*nap*	*ole*	*car*	Product group	Gene	*cam*	*jun*	*pol*	*nap*	*ole*	*car*
Complex I	*nad1*	+	+	+	+	+	+	Ribosome	*rps3*	+	+	+	+	+2	+
	*nad2*	+	+	+	+	+	+		*rps4*	+	+	+	+	+	+
	*nad3*	+	+	+	+	+2	+		*rps7*	+	+	+	+	+2	+
	*nad4*	+	+	+	+	+2	+		*rps12*	+	+	+	+	+2	+
	*nad4L*	+	+	+	+	+2	+		*rps14*	+	+	+	+	+2	+
	*nad5*	+	+	+	+	+2	+		*rpl2*	+	+	+	+	+2	+
	*nad6*	+	+	+	+	+2	+		*rpl5*	+	+	+	+	+2	+
	*nad7*	+	+	+	+	+	+		*rpl16*	+	+	+	+	+2	+
	*nad9*	+	+	+	+	+2	+	*tRNA*							
Complex III	*cob*	+	+	+	+	+2	+	*Asparagine*	*trnN*	+	+	+	+	+2	+
Complex IV	*cox1*	+	+	+	+	+2	+	*Aspartic*	*trnD*	+	+	+	+	+2	+
	*cox2-1*	+	+	+	+	+2	+	*Cysteine*	*trnC*	+	+	+	+	+2	+
	*cox2-2*	+	+	+	+	-	-	*Glutamic*	*trnE*	+	+	+	+	+2	+
	*cox3*	+	+	+	+	+	+	*Glutamine*	*trnQ*	+	+	+	+	+2	+
Complex V	*atp1*	+	+	+	+	+2	+		*trnG*	+	+	+	+	+	+
	*atp4*	+	+	+	+	+2	+	*Histidine*	*trnH*	+2	+2	+2	+	+4	+
	*atp6*	+	+	+	+	+2	+	*Isoleucine*	*trnI*	+	+	+	+	+2	+
	*atp8*	+	+	+	+	+	+	*Lysine*	*trnK*	+	+	+	+	+2	+
	*atp9*	+	+	+	+	+	+	*Methionine*	*trnM*	+	+	+	+	+2	+
Cytochrome c	*ccmB*	+	+	+	+	+2	+	*fMethionine*	*trnfM*	+	+	+	+	+2	+
	*ccmC*	+	+	+	+	+	+	*Proline*	*trnP*	+	+	+	+	+2	+
	*ccmFN1*	+	+	+	+	+	+	*Serine*	*trnS*	+3	+3	+3	+3	+6	+3
	*ccmFN2*	+	+	+	+	+2	+	*Tryptophan*	*trnW*	+	+	+	+	+2	+
	*ccmFC*	+	+	+	+	+	+	*Tyrosine*	*trnY*	+	+	+	+	+2	+
Other ORF	*tatC*	+	+	+	+	+2	+	*rRNA*	*rrn5*	+	+	+	+	+	+
	*matR*	+	+	+	+	+2	+		*rrn18*	+	+	+	+	+	+
	*orf222/4*	-	-	+	+	-	-		*rrn26*	+	+	+	+	+2	+

+ denotes present, - denotes absent. Gene copy number is shown after +. Data for *pol *and *nap *are from our previous report and Handa's results [6], respectively.

Previous studies have reported physical maps of the *cam *[18,24] and *ole *[17,24] mitochondrial genomes. We found that the length of *cam *sequence is almost the same as that obtained from the physical map (219.7 kb vs. 218 kb), but because of a large duplication, the length of the *ole *mitotype is 360.3 kb, much larger than that previously reported from physical mapping (219 kb). A reasonable explanation for the discrepancy may be that the large repeats of *ole *are difficult to detect by physical mapping.

### Repeats

**Figure 2:**
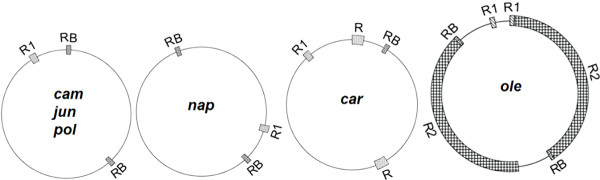
**Large repeats exist in the six mitotypes**. RB, R1, R, and R2 denote repeats of more than 2 kb. RB and R1 are shared by the six mitotypes, but their copy numbers vary.

Large repeats are a cause of the formation of the multipartite structure of the *Brassica *mitochondrial genome, including one master circle and two smaller subgenomic circles, through reversible homologous recombination [18]. A pair of large (2,427 bp) repeats, called the RB repeats, identified in *pol *and *nap *is also found in the *cam*, *jun*, and *ole *mitotypes, but only one copy of RB is found in the *car *mitotype (Figure 2). Two other pairs of large repeats, R1 and R2, are also found in *ole*; R2 is an mtDNA fragment 141.8 kb in size, and R1 carries two exons of the *nad5 *gene and is 3,605 bp in size. One copy of R1 is found in all other mitotypes except the *ole *mitotype. *Car *contains two copies of the 6,580 bp R repeats that are not homologous to the repeats of RB, R1 (Figure 2), one copy of RB, and one copy of R1. Therefore, the multipartite structures of *cam*, *jun*, *pol*, and *nap *might result from the same large RB repeats, and the multipartite structure in *car *might result from R repeats, but the multipartite structure in *ole *is complex and is not predicted because it has multiple large repeats. The sizes of multipartite circles for the other five mitotypes except *ole *predicted by this inference are given in Additional file 1, Table S1.

Tandem repeats exist in all six mitotypes. Frequency distribution analysis of the six mitotypes shows that the size of tandem repeat is mainly between 11 and 40 bp, and median of the number of repeats is mainly 2-3 (Additional file 1, Tables S2 and S3). Short repeats range from 30 to 500 bp in length with a total number of approximately 190, accounting for roughly 5% of the entire mitochondrial genome.

**Figure 3:**
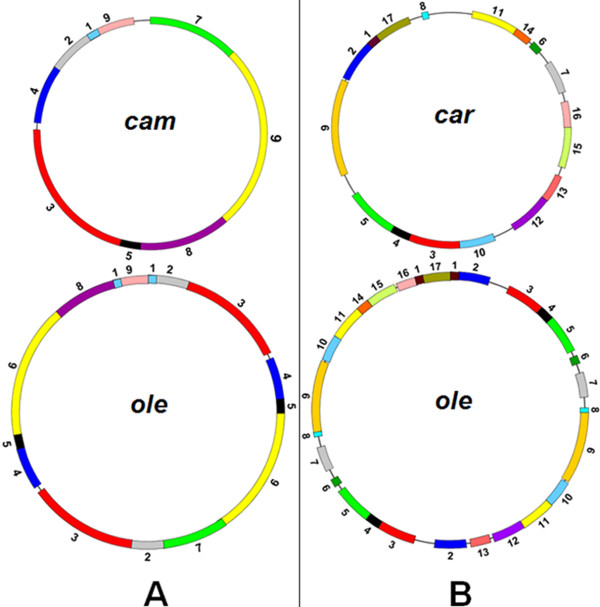
**Rearrangements of *Brassica *mitochondrial genomes**. Syntenic regions > 2 kb are shown. (A) Rearrangement of the *cam *mitochondrial genome with the *ole *mitotype as a reference. (B) Rearrangement of the *car *mitochondrial genome with the *ole *mitotype as a reference. The numbers refer to the syntenic regions derived from a paired comparison. Highly or completely homologous regions are indicated by color.

**Figure 4:**
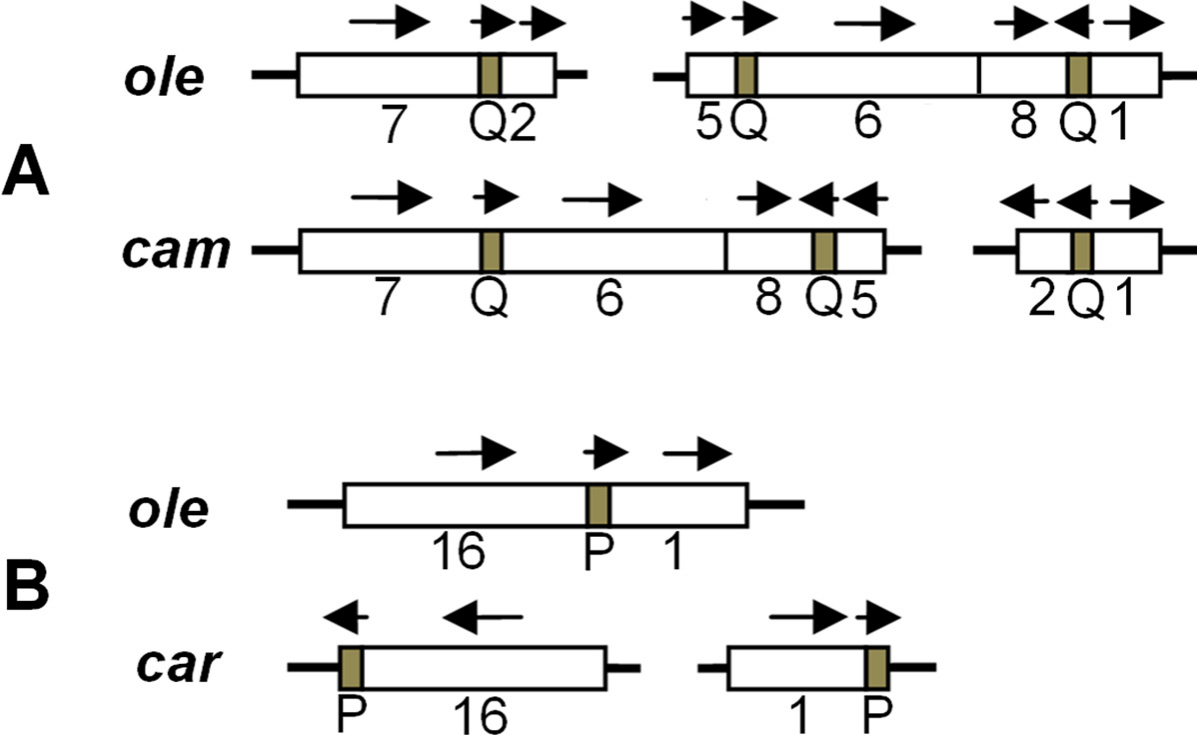
**Short repeats associated with changes in the mitochondrial genome of *Brassica***. The orientation of the sequence is shown by an arrow. (A) Repeat Q is possibly related to the influence of the rearrangement of syntenic regions 7, 2, 5, 6, 8, and 1 of the *ole *and *cam *mitotypes. (B) Repeat P may be related to the rearrangement of regions 16 and 1 in the *ole *and *car *mitotypes.

Short repeats are closely related with an irreversible re-organization of the *Brassica *mitochondrial genomes [7,25,26]. The short repeats are uniformly distributed in the six mitochondrial genomes, as detected by the Kolmogorov-Smirnov method [27]. This may imply that there are prerequisites for the irreversible rearrangement. To investigate the relationships of syntenic region rearrangements, syntenic regions were numbered (defined in Figure 3). We found that some rearrangements of numbered regions are associated with short repeats. For example, Figure 4A shows that in the *cam *and *ole *mitotypes, syntenic segments 7, 2, 5, 6, 8, and 1 were rearranged together with sequence direction changes, and the recombinations that resulted in these rearrangements are related to the short repeats marked Q, which are made up of approximately 100 bp repeats within the neighboring segment. Another typical example is the short repeat of about 70 bp (P): the separated segments 16 and 1 that contain the short P repeat in the *car *mitotype were recombined into the rearranged form in *ole *omitting the P repeat (Figure 4B). These findings are evidence for an association of short repeats with rearrangements.

A 141.8 kb segment duplication in the *ole *mitotype was the main factor influencing the differences in the number of functional genes between *ole *and the other five mitotypes. This large duplicated 38 genes with known functions and several partial exons, including three exons of *nad1*, three exons of *nad2*, and three exons of *nad5*. This partial duplication of the mitochondrial genome and the resulting dramatic change in the copy number of genes and ORFs of *B. oleracea *suggests that the role of *ole *mitotype is different from that of the other five *Brassica *mitotypes in cytotype-nuclear genotype interactions or plant-environment interactions.

### Inheritance of mitochondrial genomes in *Brassica*

By comparing the complete sequences, we found that the *cam *and *jun *mitotypes have the same genome organization and are closely related at the nucleotide sequence level. According to U's triangle for the cytological relationship between *Brassica *species, *B. juncea *is derived from the offspring of an interspecific cross between *B. rapa *and *B. nigra; *thus, the *cam *mitotype of *B. rapa *can be inferred to have been transmitted into *B*. *juncea *without a significant genomic change. Similarly, the *pol *mitotype in *B. napus*, which is postulated to be derived from the offspring of an interspecific cross between *B. rapa *and *B. oleracea *according to U's triangle, has only one region different from *cam*, suggesting that *pol *also inherited the *cam *mitotype, with an evolutionary modification. At the modification position, *pol *has a 4.4-kb insertion and *cam *has an 813-bp insertion, suggesting that *pol *was derived from *cam *at this location through evolutionary modifications resulting in a 813-bp sequence deletion and 4.4-kb sequence insertion. The relationship between *pol*, *jun*, and *cam *shows that the cytoplasmic origin of plant species can be traced because the mitochondrial genome is conserved. On the other hand, this pattern of mitotype inheritance in plants, for which we are presenting the first evidence, indicates that the evolution of new composite species is not always accompanied by sudden mitochondrial genome changes, or that new species may inherit maternal mitochondrial genomes with a few insertions/deletions (indels), as shown in the *pol *mitotype. This mitotype inheritance mechanism might be involved in other composite plant species formed by chromosomal doubling or interspecies hybridization.

### Mitochondrial genome restructuring

Relative to three highly similar mitotypes (*cam*, *jun*, and *pol*), *nap*, *ole*, and *car *were found to be restructured mitotypes. The genomic structure of the *ole *mitotype is different from that of *cam *(Figure 3A), and it can be inferred that, relative to *cam*, the genomic structure of *ole *has experienced at least four recombination events. The recombination events and a large duplication (141.8 kb) (Figure 2) made the *ole *mitotype distinct from the other five mitotypes. Relative to *ole*, the *car *mitochondrial genome has as many as 17 syntenic regions (Figure 3B), suggesting that the *car *mitotype restructuring is more complex. The number of recombination events in the process of mitochondrial genome formation in *car *is difficult to estimate, because there are so many syntenic regions.

The similarities between the syntenic regions (Additional file 2) of the six *Brassica *mitotypes are very high, with the lowest nucleotide identity being 87% and most of the nucleotide identity being more than 99%, indicating that the syntenic regions are consistently conserved in *Brassica*.

### Mitotype divergence

**Figure 5:**
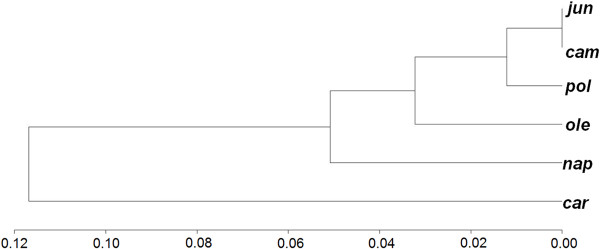
**Clustering tree of *Brassica *mitotypes**. It's according to the distance based on indels > 400 bp and SNPs (see Additional file 1, Tables S4 and S5).

Structural comparisons between two mitotypes help to identify indel sequences present in one mitochondrial genome and absent (or deleted) in another. Indels frequently found in both coding and non-coding gene regions of plant mitochondrial genomes can provide evolutionary clues [8,28]. To illustrate the genomic differences, we summed the length of indels found in a mitochondrial genome comparison and defined a dissimilarity indicator to measure the divergence between two mitochondrial genomes using indels and single nucleotide polymorphisms (SNPs) between them. Cluster analysis of the six mitotypes showed that *cam *and *pol *are in one class, *ole *slightly diverges from the *cam*-*pol *class, and *nap *and *car *have diverged the furthest from the *cam*-*pol *group (see Figure 5 and Additional file 1, Tables S4 and S5).

Combined indel analysis of multiple mitochondrial genomes showed that all insertions in the *cam*, *jun*, *pol*, and *ole *mitotypes can be aligned with other mitotypes. However, for some insertions in the *nap *and *car *mitotypes, matching sequences with high homology could not be found in other *Brassica *mitotypes. These unmatched insertions account for 2.92% of the *nap *mitotype (one insertion larger than 2 kb) and 10.36% of the *car *mitotype (11 insertions).

### Variation of open reading frames with unknown functions

Using the ORF Finder software at NCBI, the *cam, pol*, *nap*, *ole*, and *car *mitotypes were found to contain 44, 45, 46, 44, and 36 species of ORFs with unknown functions, respectively, indicating that the numbers of ORFs in the six mitotypes are different (*jun *has an ORF constitution identical to *cam*). On the other hand, the six mitotypes share 20 ORFs with unknown functions: *orf100b*, *orf101a*, *orf101b*, *orf104*, *orf106b*, *orf108b*, *orf109*, *orf113a*, *orf115a*, *orf115c*, *orf115d*, *orf116*, *orf119*, *orf120*, *orf135*, *orf146*, *orf159*, *orf161*, *orf257*, and *orf448*. Another 34 ORFs sequences are not possessed consistently by all of the six *Brassica *mitotypes (Table 3) (defined here as polymorphic ORFs, present in one mitotype in complete form, and completely absent or present in partial or mutated form in other mitotypes). This result suggests that the inclusion of ORFs with unknown functions in different mitotypes are very different.

**Table 3:** Function-unknown ORFs predicted by ORF Finder in six *Brassica *mitotypes

ORF	*cam*	*pol*	*nap*	*ole*	*car*	class	ORF	*cam*	*pol*	*nap*	*ole*	*car*	class
*orf101c*	+	+	+	2+	-	GR	*orf106a*	-	-	+	2+	+	M
*orf265*	+	+	*orf261*	orf266*(2+)	*orf277*	GR	*orf108a*	+	+	+	+	-	M
*orf305*	+	+	orf322*	*orf311*(2+)	orf310*	GR	*orf110*	+	+	+	2+	-	M
*orf101d*	+	+	+	2+	-	ID	*orf112*	+	+	+	2+	-	M
*orf117b*	-	-	+	2+	+	ID	*orf113b*	+	+	+	2+	*orf170*	M
*orf122*	+	+	-	2+	-	ID	*orf114*	+	+	+	-	+	M
*orf124*	-	-	-	-	+	ID	*orf115b*	+	+	+	2+	-	M
*orf128*	+	+	+	2+	-	ID	*orf117a*	+	+	+	+	-	M
*orf132*	+	+	-	2+	-	ID	*orf118*	+	+	*orf124*	*orf120a*	*orf123a*	M
*orf138a*	-	-	-	-	+	ID	*orf123*	+	+	+	2+	-	M
*orf188*	-	-	+	-	-	ID	*orf125*	+	+	+	2+	*orf108c*	M
*orf195*	+	+	+	-	-	ID	*orf128a*	-	-	-	-	+	M
*orf222^#^*	-	*orf224*	*orf222*	-	-	ID	*orf131*	-	-	-	-	+	M
*orf100a*	+	+	+	2+	*orf100c*	M	*orf147*	+	+	+	+	*orf141*	M
*orf101e*	+	+	+	2+	-	M	*orf164*	+	+	+	+	-	M
*orf101f*	+	+	+	+	-	M	*orf165*	-	-	-	-	+	M
*orf105*	-	-	-	2+	-	M	*orf293*	+	+	*orf286*	-	*orf288*	M

**Total number of function-unknown orf in six mitotypes**								24 (44)	25 (45)	26 (46)	24 (44)	16 (36)	

Each line presents the homologous ORFs. +, present; -, absent; GR, gene rearrangement; ID, insertion/deletion; M, mutation. ORF copy numbers are shown ahead of +. Class shows the cause of change in each line.

* indicates an exception for class and that the cause is mutation.

^# ^*orf222 *and *orf224 *are the same as Table 1.

Data for *pol *and *nap *are from our previous report and Handa's results [6], respectively. The total numbers outside parentheses show the changing ORF for each mitotype; the number in parentheses contains the ORFs that exist consistently in the six mitotypes. The total number of ORFs in *ole *is not plus the multiple copies, if containing the copies the total number is 73.

Genome rearrangement may result in loss of ORFs through a DNA break within an ORF, which results in loss of the ORF or generation of a novel ORF through the recombination of the broken sequences with others. In our analysis, three polymorphic ORFs were found to be due to genome rearrangement (Table 3). For example, the breakage of *orf101c *at position +108 due to genome rearrangement has led to the loss of this ORF in *car*, and the *orf265 *sequence in *nap *was lost in the course of rearrangement, generating *orf261 *[7]. In *car*, breakage of the *orf265 *sequence at position +463 was followed by recombination of its terminal sequence with another sequence, resulting in *orf277 *of the *car *mitotype.

Ten polymorphic ORFs were caused by indels (Table 3), indicating that indels had a direct influence on ORF variation. *Orf101d *and *orf128 *are absent in *car*, *orf117b *is absent in *cam *and *pol*, *orf122 *and *orf132 *are absent in *nap *and *car*, and *orf124 *and *orf138a *exist only in *car*. The region including *orf224 *in *pol *and *orf188*, *orf222 *in *nap *is contained in an insert region, as shown in comparison of the two mitotypes (Additional file 2).

Twenty-one out of the 34 polymorphic ORFs resulted from nucleotide mutations (Table 3), suggesting that mutations have a more important role than other evolutionary events in mitotype formation in *Brassica*. There are a total of 105 mutation sites in the 21 polymorphic ORF sequences, of five mitotypes, of which 67 sites are frame-preserving mutations although the length of ORF is changed, three sites are single nucleotide mutation sites resulting in premature stop codons, four are triplet indels of less than 52 nucleotides in length (in *orf120a, orf108c*, *orf286*, and *orf288*), which cause length variation among the homologous ORFs, and the other 31 sites are non-triplet indels, including two sites of 1 bp indels, six sites of 2 bp indels, four sites of 4 bp indels, five sites of 5 bp indels, and 14 sites of indels more than 5 bp. For example, *orf118 *is incomplete because of several nucleotide mutations in *car*. In the *ole *mitotype, mutation of nucleotides TC to AA at positions +188 and +189 in *orf120a *occurred together with a AGTATT insertion at + 212 to generate *orf120a*.

Little is known about the roles of the predicted ORFs, because limited studies have been carried out on them. Only one ORF, the CMS-related *orf222 *(or its homolog *orf224*), has been demonstrated to be important, but the ORFs with unknown functions specific to *Brassica *could have a particular role in the mitochondrial activities of a particular species [29].

Given the above-mentioned numbers of polymorphic ORFs with unknown functions, the importance of the roles of the three evolutionary factors in genome formation may be arranged in the following order: mutation > segment indel > genome rearrangement. The mechanism including these evolutionary factors undoubtedly changed the mitochondrial genome structure and ORFs, but these factors influencing the *Brassica *mitochondrial genomes did not alter the constitution of the gene species (or classes) with known functions, which is almost same as the situation in *A. thaliana *[1]. This result suggests that genes with known functions in the mitochondrial genomes are not changed, and it may indicate that the genes with known functions are too important to be lost or indispensable across the long evolutionary time in the plant genus or within several closely related species.

## Discussion

### Mitotype formation in *Brassica*

In previous analyses of plant mitochondrial genomes, several evolutionary factors, such as duplication, rearrangement, indel, and mutation, have been found to be important in genome evolution [5,8,9,30]. In this study, as well as these evolutionary factors, we have shown for the first time that mitotype inheritance can explain well the formation of some of the mitotypes (*pol *and *jun*) of *Brassica*. Thus, we suggest that the *Brassica *mitotypes are derived through a mechanism including several evolutionary events, such as inheritance, duplication, rearrangement, indel, and mutation of the plant mitochondrial genome sequence. However, this is not our final conclusion on mitochondrial genome formation because this cannot explain the phenomena resulting from our syntenic region alignment.

We have inferred the origin of two mitotypes (*pol *and *jun*), but how have the other four mitotypes originated? We analyze this result as follows.

First, U's triangle can help reveal the origin of mitotypes (Figure 1). From the cytogenetics of *Brassica*, the composite species have been thought to be formed by interspecific hybridization between elementary species and following chromosome doubling, and the mitotypes of composite *Brassica *may be regarded as having been derived by inheritance from corresponding maternal parents with primary mitotypes. In the past, the maternal parents of amphiploid species were inferred to be the species with more chromosomes on basis of the crossability between two parents. For example, *B. rapa *is regarded as the ancestral female parent for *B. napus *because a cross with *B. rapa *as maternal parent and *B. oleracea *as male parent is easier to accomplish than its reverse cross, and mitotypes of *B. napus *including *nap *can be regarded as inherited from the *cam *mitotype (*B. rapa*) although this lacked evidence at the molecular level.

Second, our combined insertion alignment results show that the insertions in the *cam*, *jun*, *pol *and *ole *mitotypes can be aligned with other *Brassica *mitotypes. This result implies that, if we regard cruciferous species with bigger mitochondrial genomes than most *Brassica *as ancestral species, the *Brassica *mitotypes can be inferred to have evolved from a common ancestral parent with all the mitochondrial genome information of *Brassica *mitotypes, and which existed after the so-called mitochondrial genome expansion of angiosperms [31]. The indels in *Brassica *mitotypes may be traces of deletions from the common ancestral parent. If we do not accept this hypothesis, then we must postulate that the insertion sequences were obtained through horizontal transfer or transfer from the nuclear genome [32,33]. The possibility that insertion sequences are transfers from the nuclear genome can be ruled out because none of them can be aligned with the nuclear DNA in a search of NCBI sequence databases. As for insertion sequences from horizontal transfer between mitochondria, the concept is seemingly logical but currently lacks supporting evidence. In addition, the so-called transfers have been considered to be random transfers, so we cannot conceive why the same insert sequences are found in different mitotypes. Thus, we conclude that there has been some genome compaction during the process of mitochondrial genome evolution.

Third, there are two mitotypes identified in *B. napus*, the *nap *and *pol *mitotypes [7,18]. The *nap *mitotype, discovered by Shiga and Thompson [34,35], exists popularly in natural rapeseed germplasm population with usually male fertile phenotype in most genetic backgrounds. The *pol *mitotype, which is sparse in natural rapeseed germplasm, resulting in cytoplasmic male sterility with easily-found restorer lines, was discovered first by Fu [36], has been widely used in to generate heterosis. No other natural mitotype apart from these two mitotypes of *B. napus *has been identified. Also, no other natural mitotype for the elementary species of *Brassica *apart from those studied here has been identified, and thus how the *nap *mitotype has been inherited appears to remain unsolved. Nevertheless, the *nap *mitotype might have been inherited from an unidentified or lost mitotype of *B. rapa*, which has very rich germplasm, and then experienced mitochondrial genome evolution events as mentioned above. Similarly, the formation of the *car *mitotype found in the composite species *B. carinata *can be explained by the same mechanism as that proposed for the *nap *mitotype. Our previous results [7] demonstrated that the *nap *and *pol *mitotypes coexist in *B. napus*: the *pol *cytoplasm consists mainly of the *pol *mitotype, and the *nap *mitotype is the main genome of *nap *cytoplasm. In this case, the inference that the maternal parent of *nap *mitotype is *B. rapa*, is further supported because the coexistence does not contradict the genome compaction hypothesis. Mitotype coexistence has also been demonstrated in other plant species, such as *Phaseolus vulgaris *[37,38]. Whether mitotype coexistence can be regarded as vestige of ancestral maternal mitochondrial genome compaction needs to be further explored, but the compaction may be a cause of mitotype diversification for the different species with different nuclei (speciation) as a result of adaptation of each plant species to internal and external environment.

**Figure 6:**
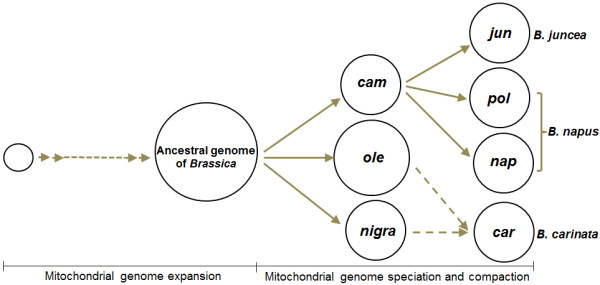
**Hypothesis regarding the evolution of six cultivated *Brassica *mitotypes**. Diverse *Brassica *mitotypes are hypothesized to have evolved from an expanded ancestral parent mitotype and formed through mitochondrial genome speciation and compaction. *Jun *(*B. juncea*) is derived from the *cam *mitotype and *pol *and *nap *from a primary mitotype very similar to the *cam *mitotype, without deletion of the CMS-related *orf224 *gene region (4.4 kb) or its homolog *orf222*. The maternal mitotype from which *car *is derived is unclear (dotted lines). Three mitotypes for the elementary species are also hypothesized to be compaction forms from the large ancestral mitotype.

Finally, from the above analysis, mitotypes for elementary species of *Brassica *can be regarded as the products of condensation or compaction of a bigger ancestral mitochondrial genome accompanied by long-term evolution history changes that may be necessary for adaptation to their environments, or be random evolutionary events such as mutation. Composite species may be inferred to have been derived from maternal mitotype inheritance and the following modification of maternal mitotype (Figure 6).

Additionally, intergenic regions of the mitochondrial genome were not found to be conserved in other studies [30,39], but our results on intergenic regions seem to be inconsistent with this conclusion. A reasonable explanation is that the mitotypes in our analysis belonging to the same genus in which closely related species were formed through genome compaction. In previous studies, either the analyzed materials were not as closely related as in our study, or the origins of the indels (the so-called non-conserved regions in other studies) were not elucidated. Our results may be an addition to the evolutionary theory of the mitochondrial genome.

### Evolutionary pattern of plant mitochondrial genomes

Mitochondrial genomes are considered to be have evolved monophyletically from a eubacterial ancestor [31,40]. The evolutionary trends are different between animals and plants over their long evolutionary history. The trend in animals is towards a further compaction of the mitochondrial genome through loss of genes and intergenic spacers, as exemplified in *Homo sapiens *and *Metridium senile *[41-43]. Conversely, the mitochondrial genomes in plants and fungi have experienced mtDNA expansions, including increases in size, primarily by acquisition of a large amount of apparently non-coding DNA of currently unknown origin and function [41,43]. This description is summarized as the serial endosymbiosis theory, which remains unrefuted [31,41].

Nevertheless, our research with *Brassica *as a phylogenetic model clade has demonstrated that a compaction mechanism has been involved in plant mitotype evolution. This phylogenetic pattern within a *Brassica *clade coincides well with the properties of the animal clade. Given this conclusion, one difference in the evolutionary mechanism between animals and angiosperms may be that the mitochondrial genome has undergone an expansion process in angiosperms but not in animals.

### Mitotype effects

Rapeseed breeders pay much attention to cytoplasmic effects. From our results, the *jun *and *cam *mitotypes do not have the CMS-related *orf222 *gene found in *nap *or the homologous CMS-related *orf224 *found in *pol*, and other genes with known functions are almost the same as in the *nap*, *pol *or *jun *mitotype, so *jun *and *cam *may probably be beneficial to breeding of conventional varieties of *B. napus *because the CMS-related gene is usually regarded as being noxious to plants [44]. The fact that the amphiploid *B. juncea *is more tolerant to stressful environments than *B. napus *[21] is probably related to the mitotype differences between *B. juncea *and *B. napus*. Potential uses of the *ole *and *car *mitotypes in *Brassica *crop breeding need to be explored.

## Conclusions

This study has compared the sequences of six *Brassica *mitotypes. The *pol *mitotype of *B. napus *and the *jun *mitotype of *B. juncea *are found to be an inherited form of the *cam *mitotype with less modification. This result may have implications for the mitochondrial genome origin of other composite plant species formed by chromosomal doubling or interspecific hybridization. Sequence relationship analyses showed that a mechanism of genome compaction and inheritance has been involved in *Brassica *mitotype evolution. Our study suggests a mechanism of mitochondrial genome formation in *Brassica *including evolutionary events such as inheritance, duplication, rearrangement, sequence compaction (indels), and mutation.

## Methods

### Mitochondrial DNA preparation

Materials used for mitochondrial genome sequencing included four *Brassica *accessions: Suzhouqing of *B. rapa*, Jiangpu-yejiecai of *B. juncea*, 08C717 of *B. oleracea*, and W29 of *B. carinata*. The first two of these were from our germplasm deposited in the National Key Lab of Crop Genetics and Germplasm Enhancement in Nanjing Agricultural University. The 08C717 accession of *B. oleracea *was provided by the Institute of Vegetable Crops in Jiangsu Academy of Agricultural Sciences, and W29 of *B. carinata *was provided by Yanyou Wu of Jiangsu University. Methods for mitochondrial DNA extraction and purification of the four species were reported previously [7].

### Genome sequencing

The *Brassica *mtDNA was sequenced using the massively parallel GS-FLX DNA pyrosquencing platform from Roche 454 Life Sciences (Branford, CT, USA). Contigs were joined by Sanger sequencing of PCR products. The number of reads for *cam*, *jun*, *ole*, and *car *were 11,805, 11,125, 15,189, and 29,676, respectively. Total sequence data for *cam*, *jun*, *ole*, and *car *were 4,432,258 bp, 4,422,401 bp, 6,798,916 bp, and 13,381,537 bp, respectively, representing mitochondrial genome coverage ratios of 19X, 20X, 18X, and 57X, respectively. The coverage ratio for the *car *mitotype is far greater than that of the others because it was sequenced twice, as the first sequencing attempt was not successful.

### Genome annotation

Database searches were carried out using the NCBI web-based Blast service [45], and genes with e-values less than 0.001 were selected. The ORFs were used to query non-redundant databases using BLAST similarity searches, by applying a cut-off of 70% sequence identity over at least 80% of the ORF length. To define genes, ORF Finder, BLASTN, BLASTX [45], and tRNAscan-SE [46] were used. To improve identification accuracy, the mtDNA annotations were compared with other plant mitochondrial genome annotations, and all differences in coding predictions were reassessed on the basis of the choice of the start codon, length of plant mtDNA conservation, and the presence of identical motifs. In addition to unknown genes, only ORFs of at least 100 codons were annotated, using the mitochondrial genome annotations of the *nap *(GenBank: AP006444.1) and *pol *(EMBL: FR715249) mitotypes of *B. napus *as a reference.

### Mitochondrial genome comparisons

The six mitochondrial genomes were aligned using BLASTN [45]. Homologous segments > 400 bp in length were chosen. Indel, defined as a sequence present in one mitochondrial genome but absent in another mitochondrial genome, were extracted to elucidate genomic divergence. Two previously reported mitochondrial genomes for *Brassica *were combined for analysis. The dot matrix plot provided in BLASTN helped in analysis of genome restructuring. Additionally, progressive Mauve [47] was used to identify SNPs between genomes.

### Analysis of repeats

Repeats (30-500 bp) were discovered using in-house private commercial software developed by Shanghai Majorbio Bio-pharm Biotechnology Company (China). BLASTN was used to identify repeats longer than 500 bp. Information on tandem repeats was obtained using the tandem repeats finder [48]. The Kolmogorov-Smirnov method was used to test the uniformity of short repeat distribution.

### Mitochondrial genome clustering

Two main indicators were used to measure mitochondrial divergence: the number of SNPs between two genomes and the total length of indel sequences that account for differences when two mitochondrial genomes were compared. Dissimilarities among mitochondrial genome sequences were measured with the formula *d *= (2*N_SNP_*+*L_Indel1_*+*L_Indel2_*)/(*L_G1_*+*L_G2_*), where *L_G1 _*and *L_G2 _*represent the entire lengths of the two mitochondrial genomes to be compared, *N_SNP _*represents the number of SNPs found when the paired genomes were compared, and *L_Indel1 _*and *L_Indel2 _*denote the total length of insertion sequences for the two mitochondrial genomes. With this definition of dissimilarity, the UPGMA method [49] was used to cluster the mitochondrial genomes of *Brassica *species.

## List of abbreviations

bp: base pairs; CMS: cytoplasmic male sterility; indel: insertion/deletion; mtDNA: mitochondrial DNA; SNP: single nucleotide polymorphism; ORFs: open reading frames.

## Authors' contributions

SC carried out the experiments and performed sequence analysis. TY, YH, JC and JY participated in the experiments. TD, JH participated in the sequence analysis. RG conceived and supervised the work, guided sequence analysis and drafted the manuscript. All authors read and approved the final manuscript.

## Supplementary Material

Additional file 1 **Supplemental Tables S1 to S5 providing detailed analyses results**. Click here for file

Additional file 2 **The syntenic regions derived from mitotype-to-mitotype comparisons**. Click here for file
