# Supplementary material for: Mitochondrial genome sequencing helps show the evolutionary mechanism of mitochondrial genome formation in Brassica
Source: BMC Genomics. 2011 Oct 11;12:497. doi: 10.1186/1471-2164-12-497 (PMC3204307; doi:10.1186/1471-2164-12-497)
Supplement: Additional file 1 — Supplemental Tables S1 to S5 providing detailed analyses results. [file 1471-2164-12-497-S1.DOC]

**Supplementary Tables**

**Table S1.** Predicted multipartite sizes (bp) in five *Brassica* mitotypes

| **Mitotype** | **Master circle** | **Big circle** | **Small circle** |
| --- | --- | --- | --- |
| *cam* | 219747 | 137111 | 82636 |
| *jun* | 219766 | 137123 | 82643 |
| *car* | 232241 | 136493 | 95748 |
| *nap* | 221853 | 124908 | 96945 |
| *pol* | 223412 | 137132 | 86280 |

**Table S2.** Length (bp) of tandem repeat sequences

| **Species** | **1-10** | **11-20** | **21-30** | **31-40** | **>40** |
| --- | --- | --- | --- | --- | --- |
| *cam* | 1 | 9 | 4 | 3 | 0 |
| *jun* | 1 | 9 | 4 | 3 | 0 |
| *ole* | 1 | 17 | 9 | 6 | 0 |
| *car* | 0 | 11 | 5 | 3 | 1 |
| *nap* | 1 | 11 | 4 | 2 | 2 |
| *pol* | 1 | 11 | 4 | 2 | 2 |

**Table S3.** Copies of tandem repeat sequences

| **Species** | **<2.0** | **2.0-3.0** | **>3.0** |
| --- | --- | --- | --- |
| *cam* | 1 | 15 | 1 |
| *jun* | 1 | 14 | 2 |
| *ole* | 2 | 30 | 1 |
| *car* | 0 | 19 | 1 |
| *nap* | 3 | 14 | 3 |
| *pol* | 3 | 14 | 3 |

**Table S4.** SNPs and indels for each genome pair

|  | ***cam*** | ***jun*** | ***pol*** | ***nap*** | ***ole*** | ***car*** |
| --- | --- | --- | --- | --- | --- | --- |
| *cam* |  | 8 | 17 | 160 | 75 | 809 |
| *jun* | 0 |  | 20 | 156 | 78 | 854 |
| *pol* | 5258 | 5258 |  | 197 | 103 | 817 |
| *nap* | 19674 | 19674 | 17215 |  | 210 | 750 |
| *ole* | 10395 | 10395 | 16466 | 29496 |  | 704 |
| *car* | 41081 | 41081 | 44341 | 50734 | 51562 |  |

Upper triangle is SNPs of each genome pair. Lower triangle is the indel of each genome pair.

**Table S5.** Distance between each genome pair

|  | ***cam*** | ***jun*** | ***pol*** | ***nap*** | ***ole*** |
| --- | --- | --- | --- | --- | --- |
| *jun* | 3.64048E-05 |  |  |  |  |
| *pol* | 0.011941727 | 0.011954672 |  |  |  |
| *nap* | 0.045276986 | 0.045256615 | 0.039547236 |  |  |
| *ole* | 0.018180752 | 0.018190407 | 0.028563548 | 0.051391289 |  |
| *car* | 0.094470785 | 0.094665301 | 0.100899149 | 0.115029047 | 0.089399335 |
